# CNCA aligns small annotated genomes

**DOI:** 10.1186/s12859-024-05700-1

**Published:** 2024-02-29

**Authors:** Jean-Noël Lorenzi, François Graner, Virginie Courtier-Orgogozo, Guillaume Achaz

**Affiliations:** 1 Université Paris Cité, Paris, France; 2 CNRS, Institut Jacques Monod, 75013 Paris, France; 3 SMILE Group, Center for Interdisciplinary Research in Biology (CIRB), Collège de France, 75006 Paris, France; 4 CNRS, Matière Et Systèmes Complexes, 75013 Paris, France

**Keywords:** Annotated genomes, Nucleotide alignment, Protein alignment

## Abstract

**Background:**

To explore the evolutionary history of sequences, a sequence alignment is a first and necessary step, and its quality is crucial. In the context of the study of the proximal origins of SARS-CoV-2 coronavirus, we wanted to construct an alignment of genomes closely related to SARS-CoV-2 using both coding and non-coding sequences. To our knowledge, there is no tool that can be used to construct this type of alignment, which motivated the creation of CNCA.

**Results:**

CNCA is a web tool that aligns annotated genomes from GenBank files. It generates a nucleotide alignment that is then updated based on the protein sequence alignment. The output final nucleotide alignment matches the protein alignment and guarantees no frameshift. CNCA was designed to align closely related small genome sequences up to 50 kb (typically viruses) for which the gene order is conserved.

**Conclusions:**

CNCA constructs multiple alignments of small genomes by integrating both coding and non-coding sequences. This preserves regions traditionally ignored in conventional back-translation methods, such as non-coding regions.

## Background

A naive nucleotide alignment of annotated genomes usually results in many frameshifts and other oddities that do not exist in protein alignments. Several methods have been developed to perform nucleotide alignments taking protein alignment into account. One approach is “back-translation”, where coding nucleotide sequences are translated into amino acid sequences that are then aligned. Corresponding codons are then aligned in a final nucleotide alignment. The web-based tool web-prank (https://www.ebi.ac.uk/goldman-srv/webprank/; [1]) is such an example. Other tools based on back-translation propose specific options like the choice of genetic codes (PAL2NAL [2], transAlign [3], RevTans [4]). Some are designed to consider cases in which frameshifts or stop codons can occur (MACSE [5, 6], PAL2NAL [2], transAlign [3]). TranslatorX [7] checks the relevance of the amino acid alignment by finding regions of uncertainties in the amino acid alignment (masked by Gblocks [8]) and reports them in the nucleotide alignment. Others are optimized for virus gene sequences (NucAmino [9], VIRULIGN [10]. To the best of our knowledge, none of these methods processes genome alignment with both coding and non-coding regions. We have thus developed CNCA (Coding / Non-Coding Aligner), a genome-wide solution that returns a full genome alignment compatible with the protein sequence alignment. The method was designed for small (up to 50 kb) homologous annotated syntenic genomes devoid of introns, such as virus genomes. It will ease the subsequent evolutionary analysis of annotated genomes.

## Implementation

CNCA is a pipeline developed in Python and R. For the alignment steps, it uses MAFFT [11]. This pipeline can be run online at https://cnca.ijm.fr/.

In addition, a standalone version is available at https://github.com/jnlorenzi/CNCA_standalone.

**Fig. 1:**
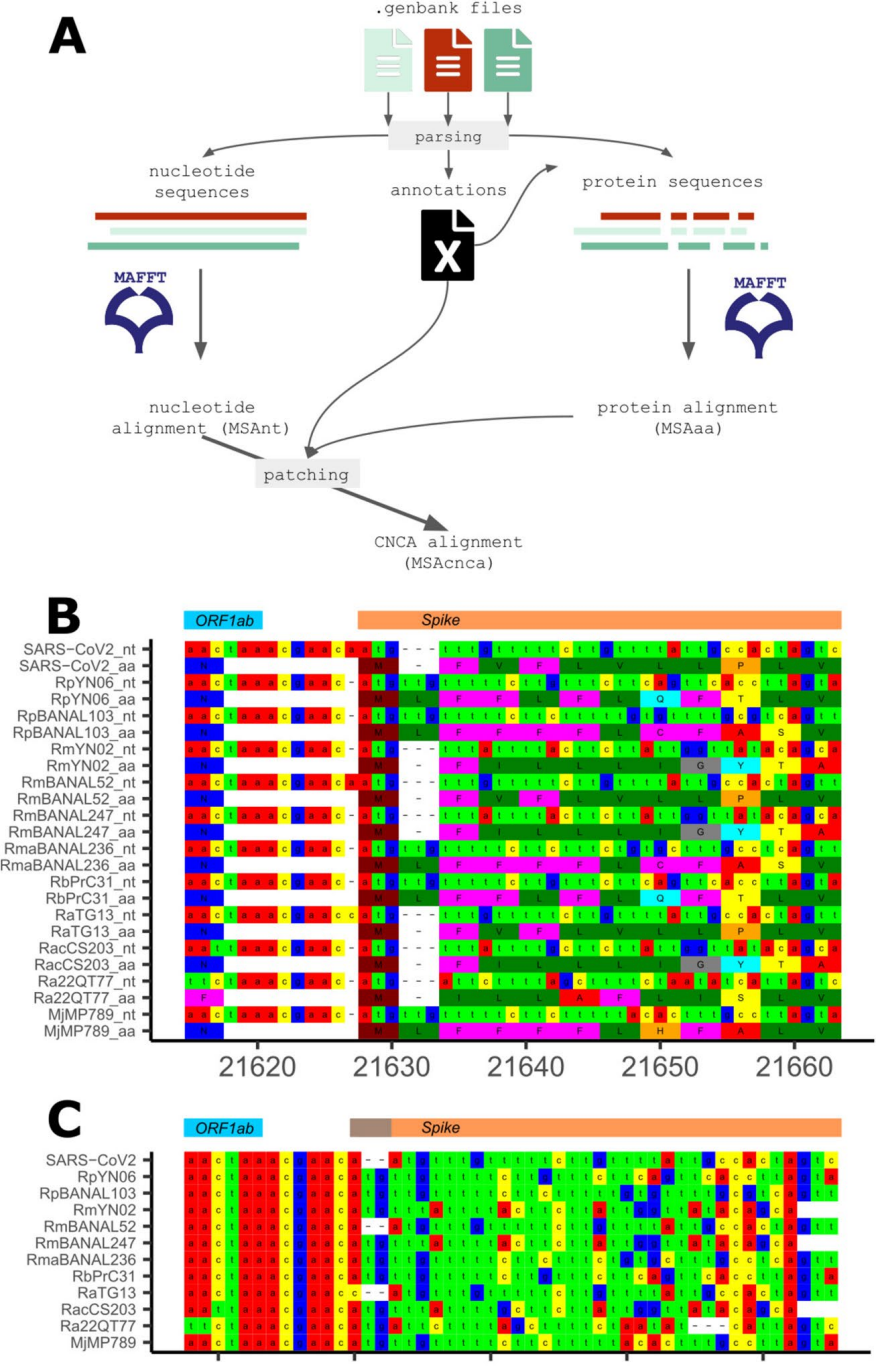
CNCA pipeline and example of use of CNCA with coronavirus sequences. **A** CNCA pipeline diagram. **B** Part of the alignment generated by CNCA for SARS-CoV-2 and 11 closely-related virus genomes. Virus names are indicated on the left. Wuhan-Hu-1 is SARS-CoV-2. _nt means nucleotide sequence, and _aa protein sequence. The region comprises the end of the *ORF1ab* coding region and the beginning of the *Spike* coding region (top boxes). Positions on the MSAnt CNCA alignment are indicated. **C** Corresponding nucleotide sequences aligned with MAFFT

CNCA takes as input two or more GenBank files of annotated genomes. To cap computation time on the server, sequences submitted via the online tool must be lower than 50 kb. It first MAFFT-aligns [11] the nucleotide (nt) sequence of all genomes and produces a Multiple Sequence Alignment (MSAnt). It then generates MSAaa, the MAFFT-alignment of the concatenations of all protein sequences. As the concatenated sequence takes protein sequence on the order of gene annotations, synteny must be conserved. Note that an alternative pipeline would have been to align each coding region individually between genomes, but this approach was not chosen for the sake of speed and simplicity. The MSAnt is then updated using MSAaa for all coding regions where both alignments are not concordant. A final MSAcnca is returned that contains no contradiction with MSAaa and thus no frameshift (Fig. 1A). We choose to implement a graphical web version of the pipeline to widen the potential users to non-experts. Results (logs and the three alignments MSAcnca, MSAnt, MSAaa in both nexus and fasta formats) are stored locally for a week. An email with a link to access the results is sent to the user at the end of the procedure.

## Results

As an illustration, we used CNCA on a dataset of 12 annotated genomes closely related to SARS-CoV-2. The whole pipeline runs in 45 min and generates an alignment compatible with current knowledge of coronavirus evolution. Figure 1B presents a fraction of the resulting alignment, from the end of the *ORF1ab* coding region to the start of the *Spike* coding region. The 1-bp indel present in the intergenic region between *ORF1ab* and *Spike* is detected by the CNCA approach, but not via a simple nucleotide alignment (Fig. 1C) or via a back-translation method (as it ignores non-coding regions).

## Conclusions

CNCA is a user-friendly and simple online tool. It can construct multiple alignments of small genomes by integrating both coding and non-coding sequences. We developed it for coronaviruses and it can also be used for other virus families and for short syntenic genetic loci in bacteria.

## Availability and requirements

Project name: CNCA.

Project home page: https://cnca.ijm.fr/

Operating system(s): Platform independent.

Programming language: Python, R, PHP.

License: MIT.

Any restrictions to use by non-academics: none.

## Data Availability

Project homepage: https://cnca.ijm.fr/; Standalone version available at https://github.com/jnlorenzi/CNCA_standalone.
